# Epidemiological features of patients infected with HCV genotype 4 in Poland: Epidemiology of HCV genotype 4 in Poland

**Published:** 2011-03-01

**Authors:** Slawomir Chlabicz, Robert Flisiak, Tadeusz W Lapinski, Oksana Kowalczuk, Alicja Wiercinska-Drapalo, Barbara Pytel-Krolczuk, Anna Grzeszczuk, Lech Chyczewski, Joanna Pancewicz

**Affiliations:** 1 Department of Family Medicine and Community Nursing, Medical University of Bialystok, Bialystok, Poland; 2 Department of Hepatology and Infectious Diseases, Medical University of Bialystok, Bialystok, Poland; 3 Department of Clinical Molecular Biology, Medical University of Bialystok, Bialystok, Poland

**Keywords:** Genotypes, Hepatitis C, Drug users

## Abstract

**Background:**

Hepatitis C genotype 4 (HCV-4) is considered to be rare outside northern Africa and southern Europe.

**Objectives:**

To describe the epidemiological characteristics of patients infected with HCV-4 in Poland.

**Patients and Methods:**

The study group included 290 patients with HCV-related chronic liver disease and intravenous drug users with HCV infection recruited in years 2002-2006 in Podlaskie region, north-eastern Poland. In all cases, HCV infection was confirmed by HCV-RNA detection by qualitative nested RT-PCR. HCV genotype was determined by 5'UTR sequencing and comparison with known genotype-specific sequences.

**Results:**

HCV 4 was found in 45 (15.5%) of 290 HCV-infected and HCV RNA-positive individuals. 60% of HCV 4 infections occurred in intravenous drug users; 51% of HCV 4-infected patients were also HIV-positive. Among 119 patients whose source of infection was other than drug use, there were 16 (10.5%) HCV 4 cases. Seven (46%) of 13 HCV 4-positive and HIV-negative patients who received combined antiviral treatment had sustained viral response.

**Conclusions:**

HCV 4 exists in eastern Poland, and the infection is frequently related to intravenous drug use and accompanied by HIV infection. HCV 4 also causes a proportion of non-drug-related HCV infections.

## Background

Hepatitis C is a major cause of end-stage liver disease in many parts of the world. Six genotypes 1-6 of hepatitis C virus (HCV) have so far been described [1]. Overall, HCV genotype 4 (HCV 4) represents approximately 20% of the global HCV infection [2]. It is common in the Middle East and sub-Saharan Africa. In Egypt, it is responsible for almost 90% of HCV infections [3], and has recently spread to several European countries [4][5][6][7]. The majority of HCV 4 infections in Egypt resulted from parenteral antischistosomal therapy while most of the Europeans acquired the infection through illicit drug use [8]. Some studies have indicated a potential link between infection with HCV 4 and higher risk of development of hepatocellular carcinoma [9]. Little epidemiological information concerning HCV 4 infection in eastern Europe is available. Our earlier report demonstrated decrease of relative proportion of genotype 1, and increase in genotype 3 over time and confirmed presence of genotype 4 in north-eastern Poland [10]. We also reported on a high prevalence of genotype 4 among HCV-infected intravenous drug users [11]. HCV 4 is considered difficult-to-treat genotype like HCV-1. Patients with HCV 4 genotype are underrepresented in large clinical trials because of the rare prevalence of this genotype in Europe and the US. In addition, antiviral treatment may be negatively affected by frequent co-infection with HIV [8].

## Objectives

The objective of this study was to further elaborate on epidemiological features of HCV genotype 4 infections in Poland, to determine factors associated with HCV 4 infection not related to drug use and to describe antiviral treatment response in patients infected with HCV 4.

## Patients and Methods

The study took place in Podlaskie voiewodship (administrative area in north-eastern Poland, with 1,200,000 inhabitants). Subjects included anti-HCV-positive individuals seen at the Department of Infectious Diseases and Hepatology of the Medical University of Bialystok and HCV-infected clients of the drug treatment center near Bialystok (Monar, Zaczerlany) in years 2002-2006. All subjects were interviewed using a structured questionnaire. The risky exposures considered in our study were intravenous drug use, transfusions of blood or blood products before 1993 (routine blood screening was introduced in Poland in 1992), incarceration and tattoos. The diagnosis of hepatitis C was based on the presence of anti-HCV antibodies (ELISA, third generation test, IMx MEIA, Abbott, Chicago, USA) and was confirmed by HCV-RNA testing (qualitative nested RT-PCR) as previously described [8][9]. HCV genotypes were determined by direct nucleic acid sequence analysis of nested-PCR products from 5'UTR, as previously described in detail [8][9]. The INNO-LIPA HCV II hybridization test (Innogenetics, Belgium) was also used in our study to confirm the results of HCV genotyping by 5'UTR sequencing for 19 patients and concordant results were obtained. The statistical calculations were performed with the use of statistical package Statistica 7.1 (Statsoft, Tulsa, OK, USA). x(2) test (with Yate's correction when appropriate) was used for analysis of differences in risk factors between groups with genotype 4 and other genotypes.

Informed consent was obtained from each participant and the study protocol was approved by the Ethical Committee at the Medical University of Bialystok. A p-value < 0.05 was considered statistically significant.

## Results

Among 290 patients with HCV infection who had HCV genotype determined, there were 45 (15.5%) patients infected with genotype 4, 146 (50.3%) with genotype 1 and 99 (34.1%) with HCV 3. The majority of patients with genotype 4 had similar characteristics to those infected with other genotypes and were males (69%) aged between 31 and 50 years of age (66.7%) poorly educated (basic education or lower secondary 55.6%) (Table 1). The mean age of patients with HCV 4 and those infected with other than HCV 4 genotype was not significantly different (34 and 36.9 years, respectively). Overall 60% of HCV 4 infections occurred in patients with prior intravenous drug use while 37.6% of infections with other genotypes were drug-related. In 119 patients whose source of infection was other than drug use, there were 16 (10.5%) HCV 4 cases. The majority (51%) of HCV 4 individuals were also HIV-positive (28.9% in case of other genotypes) (Table 1). Among 94 patients with HCV/HIV co-infection, HCV 4 was present in 23 (24.5%) individuals. Thirteen of HCV 4-positive patients who were HIV-negative and never received treatment for HCV (9 men, 4 women, mean ± SD age of 41.6 ± 11.9 years) received combined antiviral treatment (Peg-IFN α-2a 120-180 µg/week, Peg-IFN α-2b 100-150 µg/week or natural IFN α 9 MU/week with ribavirin (800-1200 mg/day based on body weight). HCV RNA was assessed at baseline, and after 3, 12 and 18 months of start of the treatment. In three (23%) patients the treatment was stopped after three months due to lack of response (defined as at least 2 log10 reduction in HCV RNA at week 12). End-of-treatment viral response (defined as negative for HCV RNA at the end of 48 week therapy) was achieved in nine (69%) patients and sustained viral response (defined as negative for HCV RNA six months after end of therapy) in six (46%) patients.

**Table 1:** Relationship between HCV 4 and socio-de0mographic features in 290 individuals with HCV infection

	**HCV genotype 4**	**genotype otherthan 4**	**HCV genotype 1**
	45 (15.5%) No (%)	245 (84.5%) No (%)	146 (50.3%) No (%)
**Male**	31 (69)	169 (69.0)	97 (66.4)
**Age** (year)			
**< 30**	11 (24)	78 (31.8)	48 (32.9)
**31–50**	30 (67)	136 (55.5)	77 (52.7)
**> 50**	4 (9)	31 (12.7)	21 (14.4)
**Drug users**	27 (60)	92 (37.6) a	45 (30.8) b
**HIV-positive**	23 (51)	71 (28.9) a	39 (26.7) b
**First year of intravenous drug use**			
**< 1980**	1/25 (4)	4/71 (6)	1/37 (2.7)
**1980–1990**	11/25 (44)	19/71 (27)	11/37 (29.7)
**> 1990**	13/25 (52)	48/71 (68)	25/37 (67.6)
**Transfusion**	6 (13)	29 (11.8)	24 (16.4)
**Tattoo**	11 (24)	52 (21.2)	31 (21.2)
**History of incarceration**	6 (13)	26 (10.6)	11 (7.5)
**Education**			
**Elementary, grammar orlower vocational**	25 (56)	98 (40.0)	69 (47.3)
**Secondary**	15 (33)	112 (45.7)	56 (38.4)
**Higher (College or university)**	5 (11)	35 (14.3)	21 (14.4)

^a^ p < 0.05 (HCV 4 vs other than 4)

^b^ p < 0.05 (HCV 4 vs HCV 1)

## Discussion

We found that 15.5% of HCV individuals in north-eastern Poland harbored genotype 4. Overall 60% of HCV 4 infections in the present study were associated with intravenous drug use and further 13% were related to transfusions. The association with drug use which was shown in our previous report [11] is in accordance with the studies from Western Europe. For example, 57% of 134 patients of French origin infected with genotype 4 were drug-related and further 18.8% resulted from transfusion [7]. In a US study, 12 (60%) of 20 patients with HCV 4 had a history of illicit drug use [12]; the drug use however, is not the major risk factor for infection with genotype 4 in Africa. More than 90% HCV 4-infected Egyptian immigrants in France had no history of drug use; their major risk factor was a history of parenteral treatment for Schistosoma mansonii in childhood or adolescence [7]. In a study of 112 patients with HCV 4 from Kuwait, researchers reported a high proportion of individuals with Egyptian nationality, but none were intravenous drug users [13]. Our group of patients with HCV 4 did not include any immigrants. HIV infection (also related to drug use) was another risk factor associated with infection with genotype 4. HCV 4 exists in 24.5% of subjects with HCV/HIV co-infection. A high prevalence of genotype 4 was previously noted in HIV and HCV co-infected injection drug users in northern Spain (32/105, 30.5%) [4]. However, other studies of HIV/HCV co-infection reported a lower prevalence of HCV 4-8.3% in France [7], 13% in Spain [6] and 14 % in the US [14]. HCV 4 also caused 10.5% of HCV infections in non-drug users. The prevalence of genotype 4 in Europe reported in other studies ranged between 2.7% (34/1239) in upper Austria-all HCV 4 patients in this study came from Egypt [15], 7.4 % in southern France [16] and 13.2% in Greece [17]. In the 1999 Polish study, there were only 38 cases of genotype 4 (2.7%) among 1385 individuals. Most of these HCV infections were probably not related to drug use [18]. In another report from Poland, 7% of patients undergoing treatment for chronic hepatitis C were infected with genotype 4 [19]. Our data indicates that the prevalence of HCV genotype 4 is increasing in Poland. Outside Europe and the Middle East, presence of HCV 4 caused 7.2% of HCV infections in 1998-2002 in some areas in India [20]. In contrast, genotype 4 represented only 4 of 899 (0.4%) of HCV infections in Japan [21]. The distribution of HCV genotypes is of particular importance, since genotypes vary in their response to treatment. In our study the sustained viral response rate to combined treatment with interferon and ribavirin was 46% (6/13). This is very similar to the sustained viral response of 43.4% reported by Roulot, et al, in France (data of 242 naïve patients treated for 48 weeks with pegylated interferon and ribavirin) [7]. Recent reviews suggest that HCV 4 may display an intermediate sensitivity to treatment, because it is more sensitive to treatment than genotype 1, but exhibits a lower sensitivity to treatment than the genotype 2 or 3 [22][23]. Our study has some limitations. First, a bias in patient selection might not represent the whole HCV epidemic in Poland. High number of drug users and HIV-infected individuals in the study may skew the prevalence of HCV 4. The treatment described included different regiments and only a small subgroup of HCV 4 patients was treated with antiviral drugs.
